# Kajal-induced Artefact Simulating a Ciliary Body Tumor on Magnetic Resonance Imaging

**DOI:** 10.4274/tjo.52323

**Published:** 2018-06-28

**Authors:** Venkatraman Indiran, L. Raguram Subha, Jagannathan Kokilavani

**Affiliations:** 1 Sree Balaji Faculty of Medicine Hospital, Ophthalmology Clinic, Chennai, India; 2 VK Clinic, Ophthalmology Clinic, Chennai, India

**Keywords:** Artifact, magnetic resonance imaging, susceptibility

**Dear Editor,**

It is well known that magnetic resonance imaging (MRI) is a boon to the field of neurological and orbital imaging but it is equally important to be aware of the various artefacts and practical issues associated with them. Here we report such an instance where we encountered an eyeball lesion in the region of the ciliary body which disappeared on more detailed evaluation. Awareness of the possibility of such pseudolesions and the reasons for their occurrence is essential to avoid misinterpretation as true pathological lesions. 

A 34-year-old female presenting with history of headache was found to have a small nodular T2 hypointense lesion with a thin hyperintense border in the medial aspect of the left eyeball in the retrolental region (Figure 1). There was blooming on the gradient images but the lesion was not seen clearly on other images. The postgraduate resident raised the possibility of a ciliary body tumor.

However, as the lesion appearance was not characteristic of any condition, I wished to see the patient in person to see if she had applied any cosmetic products. She had applied kajal (an eye cosmetic) before the MRI scan and had not removed it. We thought the observed lesion could be due to susceptibility artefact arising from the applied kajal. We rescanned the patient after asking her to wash her face and making sure that there was no kajal around her eyes. Repeat MRI scan with routine T2 and thin heavily T2-weighted sections showed no lesion in the eyeball (Figure 2). A careful ophthalmological examination with dilated pupils also ruled out a solid ciliary body mass.

Patients having MRI scans as outpatients may present for examination after applying cosmetics including eye makeup, face lotions, nail polish and hair loss concealers. Eye and face makeup products may cause artefactual distortion of the orbital contents due to the iron oxide in the pigments used to produce dark shades of makeup. Though these artefacts do not interfere with brain imaging, it precludes imaging of orbital contents if they are of clinical concern. This susceptibility artefact is usually propagated along the frequency-encoding axis of the images.^1^ Susceptibility artefacts caused by eye makeup may mimic ocular disease such as ciliary body melanoma or cyst.^2^ The susceptibility artefacts are expectedly more prominent in association with 3-Tesla MR systems than lower field strengths. Escher and Shellock^3^ in their study involving 38 different types of cosmetics on 3-Tesla MRI found that all 5 of the eyeliners, all 3 of the mascaras, 3 of the 10 eye shadows and the 1 hair concealer created small to very large artefacts which were related to the presence of iron oxide or other metal-based ingredient. 

As it is prudent to prevent these artefacts, it would be very wise to advise patients to thoroughly remove all cosmetics before they arrive for MRI exams. According to American College of Radiology guidelines, all individuals undergoing an MR procedure must remove all readily removable metallic personal belongings and devices, body piercings (if removable), cosmetics containing metallic particles (such as eye make-up) and clothing items with metallic fasteners, hooks and zippers.^4^ Though ferromagnetic detection systems have been used in screening MRI patients primarily to prevent accidents related to external ferromagnetic objects like pocket knives, a pillar-type ferromagnetic detection system may be a useful adjunct to screen patients for biomedical implants and embedded foreign bodies.^5^

We would like to emphasize the importance of removing cosmetic products from the parts of the body to be scanned by the MRI to avoid wrong diagnosis and loss of diagnostic information.

## Figures and Tables

**Figure 1:**
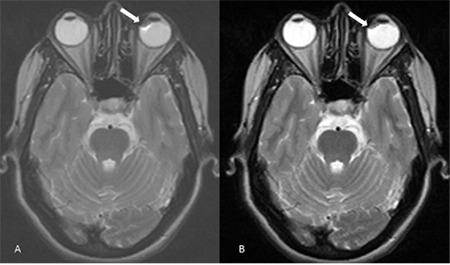
Axial T2-weighted images (A, B) show small nodular T2 hypointense lesion with a thin hyperintense border in the medial aspect of the left eyeball in the retrolental region (arrows)

**Figure 2:**
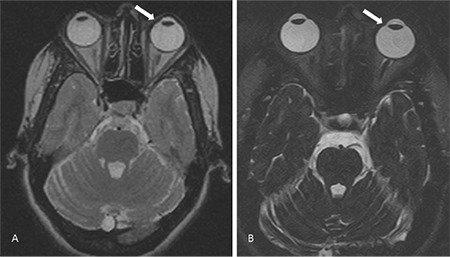
Axial 5 mm-thickness T2-weighted image (A) and thin heavily T2-weighted 1 mm section (B) showed no lesion in the eyeball (arrows)
